# Traditional Chinese medicine as a promising choice for future control of PEDV

**DOI:** 10.1016/j.virusres.2025.199572

**Published:** 2025-04-10

**Authors:** Conghao Ji, Shuxuan Li, Cunhai Hu, Tongtong Liu, Qingqing Huang, Mengyuan Yang, Mengxin Yang, Qianqian Wang, Aifang Li, Dandan Guo, Yu Huang, Sugai Yin, Shuying Feng

**Affiliations:** a Medical College, Henan University of Chinese Medicine, Zhengzhou 450046, China; b Henan Engineering Research Center for Chinese Medicine Foods for Special Medical Purpose, Zhengzhou 450046, China; c Luoyang Yiyin Industrial Co., LTD, Luoyang 471000, China

**Keywords:** Porcine epidemic diarrhea virus (PEDV), traditional Chinese medicine (TCM), pathogenic mechanism, antiviral strategies, future prospects

## Abstract

Porcine epidemic diarrhea virus (PEDV) is the major agent of the recent outbreaks of diarrhea in piglets, which has caused huge economic losses to the global swine industry. Since traditional vaccine strategies cannot provide complete protection for piglets, the development of safe, effective, and economical antiviral drugs is urgently needed. For many years, traditional Chinese medicines (TCMs) have been broadly applied for viral infectious diseases, exhibiting advantages such as abundant resources, lower toxicity, and minimal drug resistance. Many Chinese herbal monomers, single herbal extracts derived from these traditional drugs, and Chinese herbal recipes exhibit significant anti-PEDV effects *in vitro* and/or *in vivo* by targeting multiple sites and perspectives, including inhibition of the viral life cycle, anti-inflammation effects, enhancement of the host immune response, modulation of reactive oxygen species, and apoptosis. However, to date, no review has been published on the anti-PEDV effects of TCM. Therefore, this review summarizes the current control strategies for PEDV and systematically analyses the research progress of TCMs against PEDV. Furthermore, the future directions including the integration of nanotechnology and artificial intelligence with TCMs are also discussed. This review will provide a valuable reference for future studies on TCMs in antiviral research.

## Highlights

• Traditional Chinese medicines (TCMs) have been broadly applied for viral infectious diseases. This study systematically collects and analyzes recent studies on TCM monomers, single herb extracts, and recipes with anti-PEDV activity.
• Both TCM monomers and extracts from single herb, as well as TCM recipes, exhibit significant anti-PEDV activity by influencing the viral life cycle and multiple host factors.
• TCM can work synergistically with conventional antiviral drugs or vaccines to improve efficacy.
• The integration of modern technologies (such as nanotechnology and artificial intelligence) in TCM-based antiviral strategies is an inevitable trend.

## 1 Introduction

Porcine epidemic diarrhea (PED) is a severe infectious intestinal disease in pigs, characterized by symptoms such as vomiting, severe enteritis, profuse watery diarrhea, and dehydration. It is caused by the porcine epidemic diarrhea virus (PEDV) infection (Zhang et al., 2023b). PEDV can infect pigs of all age, with neonatal and suckling piglets experiencing mortality rates of 80 to 100% (Jung et al., 2020). Initially reported in Belgium and the United Kingdom in the 1870s (Debouck and Pensaert, 1979; Pensaert and de Bouck, 1978), PED later spread to various regions in Asia and the Americas from 1990 to 2010, including China, Japan, Korea, the Philippines, Thailand, Vietnam, the United States, Canada, and Mexico, albeit with relatively low incidence and mortality rates (Lee, 2015). However, since 2010, significant and severe PEDV outbreaks have occurred across Europe, Asia, and America, leading to substantial global economic losses. During 2013-2014 in the United States, around 10% of the pig population is reported to have died of PEDV resulting in estimated financial losses between 900 million to 1.8 billion (Schulz and Tonsor, 2015). PEDV has now become the most critical viral disease affecting the swine industry, posing a significant threat to the safety of pork products and the economic viability of the pig industry (Li et al., 2023a).

Several strategies have been adopted to control PEDV, including vaccination, and anti-viral drugs. Inactivated and attenuated vaccines based on classic virus strains such as CV777 are widely used, but they require with high doses and offer only short-term immunity. In recent years, several novel vaccine types such as subunit vaccines, live vector vaccines (Li et al., 2022b), nucleic acid vaccines (Wang et al., 2021), and nano-vaccines (Dhakal and Renukaradhya, 2019) have also been further developed. However, due to the genetic variation in the S1 gene of PEDV, recurrent outbreaks of PED have become more frequent in pigs that have been immunized with commercial vaccines, bringing great challenges for PEDV prevention (Su et al., 2020). Additionally, biopharmaceuticals such as monoclonal antibodies, immune sera, and egg yolk antibodies have been demonstrated effective for the prevention and treatment of PEDV, but they are limited by their high cost and complex preparation processes (Zeng et al., 2022a). Thus, it is essential to develop new strategies for anti-PEDV treatment in the future.

Due to its abundant resources, less side-effects, long history of clinical application, and minimal drug resistance, traditional Chinese medicine (TCM) has attracted increasing attention for medicinal purposes (Yu et al., 2017). Studies have reported that herbal extracts exert significant antiviral effects on human infectious viruses, including Influenza virus, human immunodeficiency virus, hepatitis B virus, and herpes simplex virus (Khwaza et al., 2018; Thomford et al., 2018; Xu et al., 2022). Additionally, some natural products or their extracts have been demonstrated to improve the production performance of animals and show antiviral activities, such as porcine reproductive and respiratory syndrome virus (PRRSV) (Chang et al., 2024), and transmissible gastroenteritis virus (TGEV) (Tan et al., 2024). In view of this, TCM holds broad potential for the control of PEDV. This review represents the first comprehensive systemic summary of TCM against PEDV. It extensively examines Chinese herbal monomers, single herbal extracts, and Chinese herbal recipes with documented anti-PEDV effects. By providing a detailed overview of current progresses, challenges, and opportunities in TCM-based anti-PEDV therapies, this review aims to promote research in PEDV control and offer valuable insights for the TCM in antiviral research, potentially accelerating the translational applications of TCM and preventing future animal husbandry and economic losses.

## 2 Etiology of PEDV

### 2.1 Genome and sbutypes of PEDV

As an enveloped, positive, single-stranded RNA virus, PEDV belongs to the genus Alphacoronavirus in the family *Coronaviridae* of the order *Nidovirales* (Lin et al., 2022). The genome of PEDV is approximately 28 kb in length and consists of seven open reading frames (ORFs) (Zuñiga et al., 2016). Among the ORFs, ORF1a and ORF1b are responsible for encoding nonstructural proteins (NSPs) that directly participate in genome replication, transcription, translation, and viral multiprotein processing, playing an indispensable role in viral genome proliferation (Ryu et al., 2021). The other ORFs encode four structural proteins and an accessory protein in the following sequence: spike (S), ORF3, envelope (E), membrane (M), and nucleocapsid (N) proteins (Lin et al., 2022). Among these proteins, the S protein play a crucial role in viral entry into host cell, where it binds to receptors on the cell surface and facilitating the subsequent fusion of the virus with the host cell membrane (Zhao et al., 2024). Based on the different gene sequences of S proteins, PEDV strains were classified into two groups, G1 and G2 genotypes (Fan et al., 2020). The G1 and G2 PEDVs were further divided into two sub-genotypes (G1-a and G1-b) and three sub-genotypes (G2-a, G2-b, and G2-c), respectively (Zhang et al., 2020). The earlier PEDV strain CV777 belongs to the G1-a genotype, and the vaccine strain DR13 belongs to G1-b. Recent studies have shown that most strains are of G2 genotype, with proportions of 100%, 95.6%, 90.2%, and 89.9% in the US, Europe, South Korea, and China, respectively (Zhang et al., 2023b). ORF3 is a co-protein involved in viral infection, and its deficiency could reduce viral virulence (Wang et al., 2012; Ye et al., 2015). The E protein can regulate PEDV infection by influencing the host immune response (Xu et al., 2013; Zheng et al., 2021). The M protein constitutes a component of the viral envelope and plays a role in the assembly and release of viral particles (Wang et al., 2020b). The N protein, which is highly conserved, significantly contributes to viral replication and transcription. Additionally, N proteins form a complex with the viral RNA genome, which serves as the core of PEDV (Zhai et al., 2023).

### 2.2 Pathogenesis

**Fig. 1:**
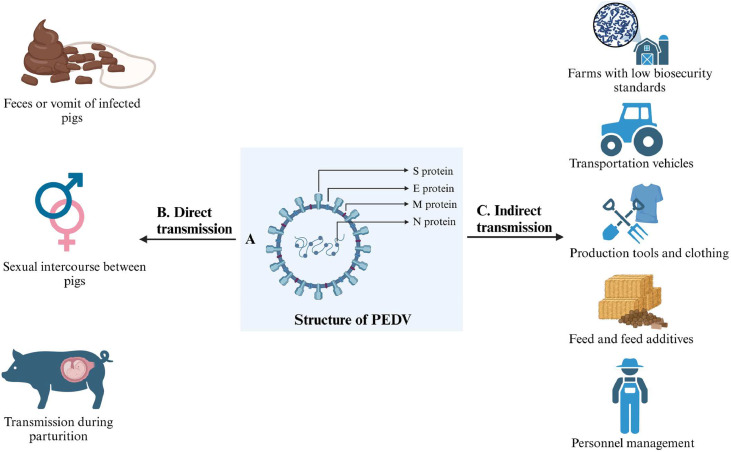
Transmission routes of PEDV. (A) Structure diagram of PEDV. Different structural proteins of PEDV are distributed on the viral envelope (S protein, E protein and M protein) and N protein. (B) Direct transmission routes. The most common direct routes of transmission contains the fecal-oral route, fecal-nasal route, sexual transmission, and vertical transmission. (C) Indirect transmission routes. The common indirect routes of transmission include the farms with low biosecurity standards, transportation vehicles, production tools and clothing, contaminated feed and feed additives, and personnel management.

PEDV transmission can occur through the fecal-oral route, fecal-nasal route, breastfeeding (Li et al., 2015), and sexual intercourse between pigs (Zhang et al., 2022). Among these, the most important is the fecal-oral route, which allows direct transmission from feces or vomit of infected pigs (Jung and Saif, 2015). PEDV enters the small intestine through the digestive tract, where the S protein of PEDV binds to specific receptors on the villous epithelial cells. Consequently, the virus fuses with the host cell membrane to invade the cells. Through the transport by dendritic cells (DCs) and lymphocytes, the virus ultimately reaches the enterocytes of the small intestine and cause lesions (Zhang et al., 2019a). The fecal-nasal route is another important pathway for PEDV transmission (Li et al., 2018; Zhong et al., 2025). DCs in the nasal submucosa can extend dendrites and enter the nasal cavity to absorb PEDV. Subsequently, dendritic cells facilitate the movement of PEDV through the mucosal barrier and delivery it to CD3^+^ T cells. These T cells then convey the virus to the intestinal lining, and result in significant atrophy of the intestinal villi and a decrease in the villus-to-crypt ratio (Liu and Wang, 2021). Consequently, the normal processes of nutrient and electrolyte digestion and absorption are impaired, leading to diarrhea characterized by malabsorption in piglets, which can escalate to life-threatening dehydration. As shown in Fig. 1, besides the aforementioned modes, the following factors contribute to the spread of PEDV: farms with low biosecurity standards (Jung and Saif, 2015), transportation vehicles (Lowe et al., 2014), humans (Kim et al., 2017b), production tools and clothing (Kim et al., 2017b), feed and feed additives (Perri et al., 2018), and transmission during parturition (Niederwerder et al., 2016).

## 3 The various strategies for controlling PEDV

### 3.1 Physical methods

**Fig. 2:**
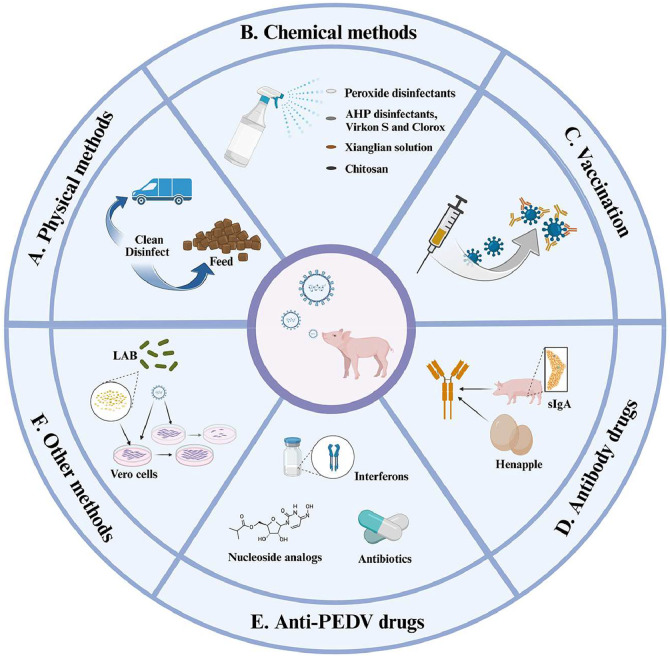
The various control strategies for PEDV infection. The current methods for control PEDV include physical methods (A. e.g., cleaning transportation vehicles and pig farm environment, contaminated feed, etc.), chemical methods (B. e.g., application of disinfectant sprays and edible disinfectant chitosan), vaccination (C. e.g., inactivated vaccines, attenuated live vaccines, genetic engineering vaccines, live vector vaccines, and mRNA vaccines), antibody drugs (D. e.g., sIgA and IgY antibodies), anti-PEDV drugs (E. e.g., nucleoside analogs, interferons and antibiotics), other methods (F. e.g., antioxidant, detoxification, antibacterial, immune modulation, and gut microbiota).

Due to the multiple transmission routes of PEDV, it is necessary to thoroughly disinfect and sterilize sources that may cause contamination (Kim et al., 2015). Physical methods are the most common, such as the strengthening sanitary management, disinfecting feed and premises, and purifying facilities and equipment, but they only provide a certain level of protective effect. Newly arrived or replaced animals, including gilts, should undergo a period of isolation to assess their health status. When raising pigs, it is critical to provide uncontaminated feed and supplements to avoid the transmission of PEDV through contact (Gordon et al., 2019; Niederwerder and Hesse, 2018). For pig farmers, the key focus should be on stringent health, feeding, and herd management practices to prevent the virus from spreading further (Kim et al., 2017c; Wang et al., 2019; Wang et al., 2018). Moreover, utilizing high-pressure washers is a common practice to maintain cleanliness in pig farms and to sanitize vehicles in contact with the farm (Fig. 2) (Baker et al., 2018).

### 3.2 Chenical methods

Chemoprevention mainly involves using compounds to inhibit PEDV activity to ensure the safety of the breeding environment and feed. For instance, treatments like super-oxidized water and hydrogen peroxide are employed to thoroughly sanitize vehicles that come in and out of the farm. This measure is taken to minimize the risk of vehicles introducing or spreading the virus on the premises. PEDV was effectively inactivated not only by super-oxidized water for 10∼90 min at room temperature (Chen et al., 2017), but also by hydrogen peroxide or peroxide disinfectants on contaminated metal (trailer) surfaces at 20°C following 30 min of exposure (Baker et al., 2017; Holtkamp et al., 2017) or at -10∼4°C following 10∼30 min of exposure (Baker et al., 2018). Additionally, it is necessary to clean and disinfect the feces of pigs on the farm. Previous studies have demonstrated that accelerated hydrogen peroxide (AHP) disinfectants (e.g., 0.5% Virkon S and 2.06% Clorox) are sufficient to inactivate PEDV in feces, which can reduce the quantity of viral RNA (Bowman et al., 2015; Holtkamp et al., 2017). Since the oral cavity is one of the main ways of PEDV transmission, contaminated nipples and feed have been suggested as vehicles of transmission for PEDV (Trudeau et al., 2016). Thus, a variety of strategies have been put forward to disinfect items entering the oral cavity of pigs and lower the potential risk of PEDV transmission via feed and its ingredients. For the piglets that need to suckle, the method of “prenatal sows immunized with PED vaccine and matched with postpartum nipple disinfection using Xianglian solution” has been developed to effectively block large-scale PEDV infection and cases in pig farms (Chang et al., 2018). For adult pigs, disinfection efforts primarily focus on feed, with wet disinfection commonly utilized. For instance, applying heat to feed at temperatures > 130°C or treating feed with electron beam irradiation at doses over 50 kg has been proven effective in reducing viral RNA and inhibiting PEDV proliferation (Gebhardt et al., 2018; Trudeau et al., 2016). Next, as a non-toxic and economical ingredient, chitosan (formed by chitin deacetylation) has been identified as an effective and safe disinfectant for inactivating PEDV. Research has found that chitosan can reduce the titer of PEDV to below 1/10,000 (under hard water conditions with 0.0067% chitosan) and 1/100 (under organic water conditions with 0.01% chitosan) of the original, thereby effectively inhibiting the virus (Bowman et al., 2015; Kim et al., 2021).

### 3.3 Vaccination

Globally, vaccine immunization remains one of the most essential preventive measures in the control of PEDV. Conventional PEDV vaccines are predominantly inactivated and modified live-attenuated vaccines (Li et al., 2020e). Research has shown that an inactivated PEDV vaccine, such as the strain KNU-141112 (G2-b), can markedly enhance the survival rates of piglets and mitigate the intensity of diarrhea (Baek et al., 2016). While inactivated vaccines offer higher safety, they tend to have lower immunogenicity compared to live-attenuated vaccines, often resulting in lower antibody levels and shorter durations of immunity. Thus, in practical PEDV control, attenuated live vaccines tend to be more effective than inactivated vaccines. Due to their advantages of difficulty to return, good safety, and easy to construct multivalent vaccines, preparation of attenuated live vaccines has become a research hotspot via genetically engineered operations, such as gene knockout, gene recombination, reverse genetics, and other methods (Deng et al., 2020; Hou et al., 2019a; Hou et al., 2019b; Lu and Zheng, 2023). Live attenuated vaccines can elicit a sustained immune response with high immunogenicity, and thus, a single immunization is usually sufficient to provide effective and long-lasting protection for piglets (Jing et al., 2022; Li et al., 2020e; Wu et al., 2019). For example, the 2-O-methyltransferase activity of PEDV nsp16 was inactivated by mutating KDKE to AAAA (i.e., K45-D129-K169-E202 mutated to A). This variant of the virus, while having diminished replication capabilities, can elicit a more robust response in terms of Type I and Type III interferons (IFNs), and has demonstrated 80% protective efficacy in piglets (Hou et al., 2019a). However, with the rise of PEDV mutant strains in recent years, vaccines designed around the G1-a genetic lineage have been insufficient to prevent outbreaks triggered by G2-b strains (Jang et al., 2023; Li et al., 2020e; Sato et al., 2018). Given the swift mutation rate of PEDV, the development of a diverse array of vaccines represents a critical pathway for future investigative efforts.

These novel vaccines have also made a significant contribution to the prevention and treatment of PEDV variants, including subunit vaccines, live vector vaccines, nucleic acid vaccines, and mRNA vaccines. A subunit vaccine uses genetic engineering to express the structural protein of PEDV, which is then processed to immunize animals, enabling them to acquire active immunity (Dong et al., 2021a). By optimizing the design of the PEDV S protein, the antigenicity of the PEDV vaccine was enhanced effectively, thereby blocking the binding of the S protein to the receptor (Lao et al., 2025). However, subunit vaccines have the disadvantages of low immunogenicity, short duration of protection, lack of cellular immune response, and high production costs. A live vector vaccine refers to the recombination of the PEDV antigen epitope into the genome of a virus or bacteria, which infects the host and continuously synthesizes the antigen protein to stimulate the body to produce specific immunity, including mucosal, humoral, and cellular immunity. They have been shown to notably elevate the levels of specific sIgA and IgG in piglets, alleviate clinical signs, curb viral shedding, and reduce intestinal damage (Dong et al., 2021a; Won et al., 2019; Wu et al., 2019). A mRNA vaccine is an innovative technology that combines molecular biology and immunology. They function by instructing cells to produce specific proteins that activate the immune system against pathogens. For instance, mRNA vaccines targeting the PEDV S protein have been developed, which have induced antibodies and cellular responses, providing protection in piglets (Zhao et al., 2024). However, the main challenges in mRNA vaccine production are cost and stability. Thus, the pursuit of novel vaccines that offer swift, potent, and long-lasting effects remains a priority for future research (Park, 2024). While vaccines are crucial for PEDV prevention, they are not infallible and do not guarantee complete immunity. Therefore, there is a need to enhance control measures with antiviral drugs to fill the gaps not covered by vaccines.

### 3.4 Antiviral drugs

#### 3.4.1 Antibody drugs

Antibody drugs used for PEDV treatment are mainly produced by refeeding and immunoglobulin yolk (IgY). The practice of refeeding involves antigen deliberate exposure in the gastrointestinal tract, resulting in the production of potent secretory IgA (sIgA) antibodies that can last approximately 7 months. As a result, newborn piglets can inherit these antibodies through maternal milk (Goede et al., 2015). Despite this, the limited safety and unstable outcomes of this method in pregnant sows, leading to some vets recommending its use in areas where lack of effective PEDV vaccines or therapeutics (Song et al., 2015). Recently, there has been growing interest in passive immunization through the ingestion of IgY from immunized chickens. This IgY preparation is part of the immune response and is characterized by its swift action, minimal impact on animals, affordability, and accessibility (Dan et al., 2018; Yokoyama et al., 1993). Results showed that none of the piglets treated with IgY died, and both the diarrhea symptoms and the amount of virus shedding were significantly reduced after the PEDV challenge (Lee et al., 2015a). For the convenience of clinical application, egg yolk antibody (EYA) powder or freeze-dried powder has been developed using spray drying and freeze-drying technologies (Li et al., 2018; Cui et al., 2012). With the prolongation of the disease course, the cure rate of anti-PEDV EYA gradually decreases. The variability in the effectiveness of orally administered IgY in practical settings could be attributed to factors such as individual differences among piglets or a decrease in the oral absorption rate of IgY (Li et al., 2014). The practical application of IgY is impacted by a multitude of factors, including the health status of piglets, the progression of the disease, breeding management practices, and the disease prevention strategies implemented on pig farms. Therefore, future efforts should be focused on developing high-titer EYAs and exploring changes in the administration route, dosage, and timing of IgY to achieve greater effectiveness. Additionally, it is important to strengthen the health management of piglets, including optimizing nutrition, environment, and hygiene conditions.

#### 3.4.2 Nucleoside analogs

Anti-PEDV nucleoside analogs are important sources of antiviral drugs, including GC376 (Kim et al., 2012; Mandadapu et al., 2012; Tiew et al., 2011; Ye et al., 2020), molnupiravir (Huang et al., 2023), and methylation of the N6 position of adenosine (m^6^A) (Chen et al., 2020a). These drugs primarily work by inhibiting viral replication or preventing virus entry, thereby reducing the symptoms and damage caused by viral infections. GC376 is capable of effectively neutralizing 50% of the viral protease activity, swiftly curbing viral replication, and demonstrating significant control over PEDV (Kim et al., 2012; Mandadapu et al., 2012; Tiew et al., 2011; Ye et al., 2020). Molnupiravir is known to reduce the PEDV load, thereby mitigating the spread of the virus (Huang et al., 2023). As chemical compounds, they are relatively straightforward to manufacture. The m^6^A RNA modification holds potential in combating PEDV infections, such as modulating host gene expression, impacting viral RNA stability, regulating host immune responses, and serving as a target for antiviral treatments (Chen et al., 2020a). These features position m^6^A modification as a promising avenue for developing new antiviral approaches. However, it often requires time to reduce viral content, and there is a high risk of recurrence after discontinuation, making it challenging to establish long-term immunological control. Therefore, m^6^A modification modulators can be used in combination with other antiviral drugs, and other immunomodulators should also be considered. Alternatively, more effective drug delivery systems can be developed to ensure that m^6^A modification modulators accurately reach cells infected by the virus, thereby enhancing the bioavailability of the drugs.

#### 3.4.3 Antibiotics and interferons

Research has identified several antibiotics with potent anti-PEDV properties, including griffithsin (Li et al., 2019), rapamycin (Ko et al., 2017), monolaurin (Zhang et al., 2021a), and salinomycin (Yajuan et al., 2024), which influence the infection and replication processes of viruses. Griffithsin, in particular, has been shown to be a potent PEDV inhibitor, preventing viral attachment and transmission between cells in Vero cells and reducing infection rates by approximately 82.8%. Further animal research is needed to validate the anti-PEDV efficacy of griffithsin (Li et al., 2019). Rapamycin has been observed to trigger autophagy in IPEC-J2 cells without compromising cell viability or the integrity of tight junctions, thus mitigating cell death induced by PEDV infection in IPEC-J2 cells. The cell survival rate in the drug treatment group is approximately 50% higher than that in the virus group (Ko et al., 2017). Since monolaurin can alleviate diarrhea, improve intestinal function, and exert a protective effect by inhibiting PEDV replication, it can be used as a drug or feed additive to control PEDV infection (Zhang et al., 2021a). Salinomycin could reduce PEDV infection by inhibiting the invasion period of virus infection in the early stage at a concentration of 1.617 μM, and it can reduce the activity of PEDV by 50% (Yajuan et al., 2024). However, prolonged antibiotic use can lead to the development of bacterial resistance, disrupt the microbiome, and raise significant biosafety concerns.

Interferons (IFNs) have powerful antiviral properties, immune regulation, and modulation of inflammatory responses. Research has shown that IFNs exhibit good antiviral capabilities against PEDV. Experiments have demonstrated that the inhibitory effect of IFN is dose-dependent, with the antiviral effect increasing as its concentration rises. Additionally, studies have found that IFNs can increase the expression of various antiviral proteins, such as myxovirus-resistant 1 and IFN-induced transmembrane proteins. Both Type I and Type III IFNs are capable of inhibiting the proliferation of PEDV *in vitro*, with Type III showing the highest antiviral activity, achieving a 45% inhibition rate of the virus in Vero cells (Chen et al., 2022a; Du et al., 2018).

#### 3.4.4 Others

In addition to the drugs mentioned above, some substances and components can also inhibit PEDV infection. Microorganism metabolites play important roles in antioxidant, antitoxic, antibacterial, immunomodulatory, and intestinal flora regulation (Chen et al., 2025). For example, microbial flora isolated from feces and saliva involved in the anti-PEDV process has been explored. *Lactobacillus acidophilus* (LAB) strains were isolated from the feces of PEDV-infected piglets, and the cell wall fractions or intracellular extracts of one of these strains had a better antiviral effect with higher cell viability and lower mRNA expression of PEDV N protein compared to unpretreated cells (Chen et al., 2022b). Another example is salivary *Lactobacillus lactis*, which could activate the FAK/PI2K/Akt signaling pathway in IPEC-J2 cells and has been found effective in preventing PEDV infection (Dong et al., 2021b). However, microbiota metabolites have a good inhibitory effect against specific viral strains or may even exhibit toxicity to host cells, limiting their broad application. Moreover, long-term use of particular microbiota metabolites may lead to the development of viral resistance, reducing their efficacy in prevention and control. Therefore, further research is needed to fully apply these substances in practical production process.

## 4 Traditional Chinese medicine (TCM) with anti-PEDV activity

### 4.1 TCM monomer

TCM monomers are active components isolated from TCM with clear structures and defined physicochemical properties, including polysaccharides, flavonoids, saponins, and more. A variety of TCM monomers have shown significant anti-PEDV activity with good safety. Their classification, mechanism, and effects on PEDV inhibition are summarized in Table 1. At least 14 phenols, 14 alkaloids, 12 flavonoids, 7 terpenoids, 3 saponins, 2 steroids, and 1 phenylpropanoid have been identified. Most of the TCM monomers were evaluated for their anti-PEDV activity *in vitro*, focusing mainly on Vero cells, PK15 cells, IPEC-J2 cells, and/or LLC-PK1 cells. Only four TCM monomers including *cepharanthine, homoharringtonine, puerarin*, and *buddlejasoponin* IVb were further examined in piglets *in vivo*. Among these, quercetin 7-rhamnoside, which belongs to the flavonoids class, showed the highest therapeutic index (TI) with >7142.8 (Choi et al., 2009a), demonstrating significant potential as a novel drug against PEDV. It is worth noting that different viral strains have varying virulence and pathogenic mechanisms, which may result in different antiviral effects for the same TCMs. Therefore, the viral strains used in the studies are also summarized in the Table 1, Table 2, Table 3.

**Table 1:** TCM monomers against PEDV.

No.	Classification	Name	IC_50_	TI	Virus strain	Dose/Administration	Mechanism	Effects	Models	References
1	Phenols	Epigallocatechin-3-gallate (ECGG)	12.39∼83.18 µM	—	HLJBY; CV777	20, 50, 100 μM	—	Inhibit PEDV attachment, entry, replication, and assembly; effect on PEDV N and ORF3 protein	Vero cells	(Huan et al., 2021)
2	Phenols	Curcumin	—	—	PEDV-LJX	4, 8, 16, 32, 64, 128, 256 μM	Enhance the antiviral effect of antiviral cytokines	Inhibit PEDV proliferation; effect on PEDV N protein	Vero cells	(Liu et al., 2023)
3	Phenols	Gallic acid	≈145 mg/L	14.58	CH/GX/2015/750A	0.02, 0.04, 0.08, 0.16, 0.31, 0.625, 1.25, 2.5, 5, 10 mg/mL	—	Inhibit PEDV proliferation	PK15 cells	(Wei et al., 2020)
4	Phenols	Ellagic acid	≈40 mg/L	65.48	CH/GX/2015/750A	0.02, 0.04, 0.08, 0.16, 0.31, 0.625, 1.25, 2.5, 5, 10 mg/mL	—	Inhibit PEDV proliferation	PK15 cells	(Wei et al., 2020)
5	Phenols	Resveratrol	≈118 mg/L	32.59	CH/GX/2015/750A	0.02, 0.04, 0.08, 0.16, 0.31, 0.625, 1.25, 2.5, 5, 10 mg/mL	—	Inhibit PEDV proliferation	PK15 cells	(Wei et al., 2020)
6	Phenols	Piceatannol	≈100 mg/L	3.74	CH/GX/2015/750A	0.02, 0.04, 0.08, 0.16, 0.31, 0.625, 1.25, 2.5, 5, 10 mg/mL	—	Inhibit PEDV proliferation	PK15 cells	(Wei et al., 2020)
7	Phenols	Hypericin	3.53±0.33 μM	—	HLJ	0.62, 1.25, 2.5, 5, 10 µM	Inhibit PEDV 3CLpro activity	Inhibit PEDV replication; effect on PEDV N protein	Vero cells	(Zhang et al., 2021b)
8	Phenols	Nordihydroguaiaretic acid	5 μM	>19.99	rPEDV (YN150 strain)-Nluc; YN150	1.25, 2.5, 5, 7.5, 10 μM	Decrease ROS production	Reduce PEDV replication	Vero cells	(Li et al., 2021)
9	Phenols	Caffeic acid phenethyl ester	1.74 μM	>57.63	rPEDV (YN150 strain)-Nluc; YN150	1.25, 2.5, 5, 7.5, 10 μM	Decrease ROS production	Reduce PEDV replication	Vero cells	(Li et al., 2021)
10	Phenols	Esculetin	5.97 μM	>16.75	rPEDV (YN150 strain)-Nluc; YN150	1.25, 2.5, 5, 7.5, 10 μM	Decrease ROS production	Reduce PEDV replication	Vero cells	(Li et al., 2021)
11	Phenols/Quinones	Emodin	2.1 μM	>50	rPEDV (YN150 strain)-Nluc; YN150	1.25, 2.5, 5, 7.5, 10 μM	—	Reduce PEDV replication	Vero cells	(Li et al., 2021)
12	Phenols	Gossypol-Acetic acid	2.9 μM	>30	rPEDV (YN150 strain)-Nluc; YN150	1.25, 2.5, 5, 7.5, 10 μM	—	Reduce PEDV replication	Vero cells	(Li et al., 2021)
13	Phenols	3′-Hydroxypterostilbene	4.29 μM	>23.29	rPEDV (YN150 strain)-Nluc; YN150	1.25, 2.5, 5, 7.5, 10 μM	—	Reduce PEDV replication	Vero cells	(Li et al., 2021)
14	Phenols	Tannic acid	4.37 μM	>22.89	rPEDV (YN150 strain)-Nluc; YN150	1.25, 2.5, 5, 7.5, 10 μM	—	Reduce PEDV replication	Vero cells	(Li et al., 2021)
			9 μg/ml	9.2	CV777	0.1, 1, 10, 100 μg/mL	—	Inhibit PEDV replication	Vero cells	(Choi et al., 2009a)
15	Alkaloids	Cepharanthine	2.53 μM	11.83	DR13att; rPEDV(DR13att)-△ORF3-GFP; ZJXS11	1, 5, 10, 20 μM; 11.1 mg/kg; Orally	—	More effective at inhibiting virus entry than other processes; effect on PEDV M and N protein; protect piglets against PEDV infection	Vero cells; Piglets	(Dong et al., 2022)
16	Alkaloids	Tetrandrine	3.5 μM	7.08	DR13att; rPEDV(DR13att)-△ORF3-GFP	1, 5, 10, 20 μM	—	Effect on PEDV M and N protein	Vero cells	(Dong et al., 2022)
17	Alkaloids	Fangchinoline	6.69 μM	4.51	DR13att; rPEDV (DR13att)-△ORF3-GFP	1, 5, 10, 20 μM	—	Effect on PEDV M and N protein; interfere with the virus entry and attachment processes; attenuate the virus	Vero cells	(Dong et al., 2022)
			3.54 μM (HLJBY)	4.8	HLJBY; CV777	1, 2, 4, 8 μM	Decrease the activity of CTSL and CTSB by suppressing lysosome acidification	Inhibit PEDV entry, not effect adsorption; effect on PEDV N protein	Vero cells	(Zhang et al., 2023a)
18	Alkaloids	(+)-Fangchinoline	4.68 μM (HLJBY)	3.41	HLJBY; CV777	1, 2, 4, 8 μM	Decrease the activity of CTSL and CTSB by suppressing lysosome acidification	Inhibit PEDV entry, not effect adsorption; effect on PEDV N protein	Vero cells	(Zhang et al., 2023a)
19	Alkaloids	Berbamine	9 μM (HLJBY)	>2.22	HLJBY; CV777	1, 2, 4, 8, 12, 16 μM	Decrease the activity of CTSL and CTSB	Inhibit PEDV entry, not effect adsorption; effect on PEDV N protein	Vero cells	(Zhang et al., 2023a)
20	Alkaloids	Berberine	—	—	HLJBY; SQ2014	100, 200 μM	Attenuate apoptosis induced by PEDV	inhibit PEDV replication and assembly; inhibit the CPE induced by PEDV	Vero cells	(Ye et al., 2019)
21	Alkaloids	Harmine hydrochloride	1.33 μM	>75.04	rPEDV (YN150 strain)-Nluc; YN150	1.25, 2.5, 5, 7.5,10 μM	—	Reduce PEDV replication	Vero cells	(Li et al., 2021)
22	Alkaloids	Harmine	1.96 μM	>51.1	rPEDV (YN150 strain)-Nluc; YN150	1.25, 2.5, 5, 7.5, 10 μM	—	Reduce PEDV replication	Vero cells	(Li et al., 2021)
23	Alkaloids	7-Ethylcamptothecin	<1.25 μM	>80	rPEDV (YN150 strain)-Nluc; YN150	1.25, 2.5, 5, 7.5, 10 μM	—	Reduce PEDV replication	Vero cells	(Li et al., 2021)
24	Alkaloids	Tabersonine hydrochloride	4.3 μM	19.23	rPEDV (YN150 strain)-Nluc; YN150	1.25, 2.5, 5, 7.5, 10 μM	—	Reduce PEDV replication	Vero cells	(Li et al., 2021)
25	Alkaloids	Tomatidine	3.447 μM	13.25	MS, YZ, SH, CV777	0.5, 2, 2.5, 4, 5, 6, 8, 10 μM	Inhibit PEDV 3CLpro activity	Decrease virus titer; Inhibit PEDV replication; effect on PEDV N and ORF7 protein	Vero cells;IPEC-J2 cells	(Wang et al., 2020a)
26	Alkaloids	Homoharringtonine	0.112 μM	50	CV777	100, 200, 500 nM; 0.05 and 0.2 mg/kg; Intramuscularly	Reduce the p-eIF4E level induced by PEDV	Inhibit PEDV proliferation; effect on PEDV N protein; drug-treated animals did not exhibit pathological change in tissues or symptoms of diarrhea and cachexia	Vero cells; Piglets	(Dong et al., 2018)
27	Alkaloids	Carbazole alkaloid Derivatives-No.7	—	—	CH/JXJA/2017; EGFP-PEDV (DR13att)	10, 20, 40 µM	—	Inhibition at the early stage of viral life cycle; effect on PEDV N protein	Vero cells	(Chen et al., 2021b)
28	Alkaloids	Carbazole alkaloid Derivatives-No.8	—	—	CH/JXJA/2017; EGFP-PEDV (DR13att)	20, 40, 60 µM	—	Inhibition at the early stage of viral life cycle; effect on PEDV N protein	Vero cells	(Chen et al., 2021b)
29	Flavonoids	Puerarin	—	—	Yunnan province strain	0.5 mg/kg; orally	Regulate IFN signaling pathway; Inhibit NF-κB pathway; Anti-inflammatory	Decrease mRNA levels of PEDV M and N genes; alleviate the decrease of growth performance in piglets	Vero cells; Piglets	(Wu et al., 2020)
30	Flavonoids	Quercetin 7-Rhamnoside	0.014±0.005 μg/ml	>7142.86	CV777	0.1, 1, 10, 100 μg/mL	—	Inhibition at an early stage of viral replication	Vero cells	(Choi et al., 2009a)
31	Flavonoids	Quercetin	1.7±0.8 μg/ml (CV777); 6.897 μM (CV777); 2.12 ± 0.04 µM (YN144) and 2.56 ± 0.62 µM (DR13)	214.8 (CV777)	CV777, YN144, DR13	0.1, 1, 10, 100 μg/mL; 6.25, 12.5, 25, 50, 100, 200, 400 µM	Inhibit PEDV 3CLpro activity	Reduce the number of PEDV-infected cells	Vero cells	(Choi et al., 2009a; Dong et al., 2018; Li et al., 2020d)
32	Flavonoids	Wogonin	58.87 μM (Vero cells) and 56.56 μM (IPEC-2cells)	8.07; 8.83	AH2012/12	12.5, 25, 50, 100 μM	Inhibit PEDV 3CLpro activity	Inhibit PEDV internalization, replication, and release	Vero cells; IPEC-J2	(Wang et al., 2023a)
33	Flavonoids/Phenols	Xanthohumol	3.865 μM (HLJBY)	>10.35	HLJBY; SQ2014; CV777	1, 2, 5, 10 μM	—	Inhibit PEDV M genes and N protein	Vero cells	(Zhao et al., 2020)
34	Flavonoids /Phenols	Apigenin	0.1±0.1 μg/ml	>370.4	CV777	0.1, 1, 10, 100 μg/mL	—	Inhibit PEDV replication	Vero cells	(Choi et al., 2009a)
35	Flavonoids /Phenols	Luteolin	0.2±0.2 μg/ml	32.7	CV777	0.1, 1, 10, 100 μg/mL	—	Inhibit PEDV replication	Vero cells	(Choi et al., 2009a)
36	Flavonoids /Phenols	Catechin	11.1±7.1 μg/ml	>9	CV777	0.1, 1, 10, 100 μg/mL	—	Inhibit PEDV replication	Vero cells	(Choi et al., 2009a)
37	Flavonoids	Licochalcone A	4 μM	>25	rPEDV (YN150 strain)-Nluc; YN150	1.25, 2.5, 5,7. 5, 10 μM	—	Reduce PEDV replication	Vero cells	(Li et al., 2021)
38	Flavonoids /Phenols	Proanthocyanidins	2.19 μM	>45.71	rPEDV (YN150 strain)-Nluc; YN150	1.25, 2.5, 5, 7.5, 10 μM	—	Reduce PEDV replication	Vero cells	(Li et al., 2021)
39	Flavonoids /Phenols	(E)-Cardamonin	2.15 μM	>46.44	rPEDV (YN150 strain)-Nluc; YN150	1.25, 2.5, 5, 7.5, 10 μM	—	Reduce PEDV replication	Vero cells	(Li et al., 2021)
40	Flavonoids /Phenols	(-)-Epigallocatechin Gallate	8.764 μM	11.3	MS	0.5, 2, 4, 6, 8, 10 μM	—	Inhibit PEDV infection	Vero cells	(Wang et al., 2020a)
41	Terpenoids	Betulonic acid	<1.25 μM	>49.52	rPEDV (YN150 strain)-Nluc; YN150	1.25, 2.5, 5, 7.5, 10 μM	Decrease ROS production	Reduce PEDV replication	Vero cells	(Li et al., 2021)
42	Terpenoids	Ursonic acid	2.13 μM	19.23	rPEDV (YN150 strain)-Nluc; YN150	1.25, 2.5, 5, 7.5, 10 μM	Decrease ROS production	Reduce PEDV replication	Vero cells	(Li et al., 2021)
43	Terpenoids /Others	Levistolide A	—	—	DR13; CV777; YN15; GDU	20, 40, 60, 80 µM	Stimulate the level of ROS and ER stress	Prevent PEDV from invading cells; inhibit PEDV replication	Vero cells; LLC-PK1 cells	(Zeng et al., 2022b)
44	Terpenoids	Gynostemma Extract	2.7 μM	>30	rPEDV (YN150 strain)-Nluc; YN150	1.25, 2.5, 5, 7.5, 10 μM	—	Reduce PEDV replication	Vero cells	(Li et al., 2021)
45	Terpenoids	Oridonin	3 μM	10.16	rPEDV (YN150 strain)-Nluc; YN150	1.25, 2.5, 5, 7.5, 10 μM	—	Reduce PEDV replication	Vero cells	(Li et al., 2021)
46	Terpenoids	Demethylzeylasteral	2.37 μM	16.27	rPEDV (YN150 strain)-Nluc; YN150	1.25, 2.5, 5, 7.5, 10 μM	—	Reduce PEDV replication	Vero cells	(Li et al., 2021)
47	Terpenoids	Tubeimoside I	4.21 μM	17.76	rPEDV (YN150 strain)-Nluc; YN150	1.25, 2.5, 5, 7.5, 10 μM	—	Reduce PEDV replication	Vero cells	(Li et al., 2021)
48	Saponins	Glycyrrhizin	—	—	HLJBY	0.1, 0.2, 0.4, 0.8, 1.0 mM	Decrease proinflammatory cytokine (IL-1β, IL-6, IL-8, TNF-α); inhibit HMGB1	Inhibit PEDV entry and replication; effect on PEDV N and ORF3 protein	Vero cells	(Huan et al., 2017)
49	Saponins	Glycyrrhizic acid-carbon dots	—	—	AJ1102	0.3 mg/mL	Upregulate IFN-stimulating genes	Inhibit PEDV propagation; effect on PEDV N protein	Vero cells	(Tong et al., 2020)
50	Saponins	Buddlejasoponin IVb	6.943 μM (AH-2018-HF1); 8.136 μM (MS)	12.18 (AH-2018-HF1); 11.03 (MS)	AH-2018-HF1; MS	0.5, 2, 4, 5, 6, 8, 10, 20 μM	Inhibit PEDV-activated NF-κB pathway	Inhibit PEDV replication and release; effect on PEDV N protein; relieve the clinical symptoms and intestinal damage	Vero cells;IPEC-J2 cells; Piglets	(Sun et al., 2022; Wang et al., 2020a)
51	Steroids	lithocholic acid	2.37 μM	>42.12	rPEDV (YN150 strain)-Nluc; YN150	1.25, 2.5, 5, 7.5, 10 μM	Decrease ROS production	Reduce PEDV replication	Vero cells	(Li et al., 2021)
52	Steroids	Ergosterol peroxide	20.54 μM	15.05	CV777	50, 100, 200, 300 μM	Inhibit apoptosis	Inhibit PEDV internalization, replication and release	Vero cells	(Liu et al., 2022)
53	Phenylpropanoids	Coumarin	47.4 μg/ml	4.8	CV777	0.1, 1,10, and 100 μg/mL	—	Inhibit PEDV replication	Vero cells	(Choi et al., 2009a)

“—” indicates no report.

### 4.2 TCM single herbal extracts

Due to the diverse range of active ingredients in TCM, single herbs or their extracts are often applied in treating viral diseases, such as *Hypericum japonicum* (HJ)*, Pogostemon cablin, Aloe, Alpiniae oxyphyllae, Pennisetum purpureum*, etc*.* (Table 2). For instance, six days before PEDV infection in piglets, the single TCM herb HJ was given daily throughout the experiment. The findings indicated that the PEDV-infected group without HJ treatment exhibited severe manifestations, including sudden vomiting and watery diarrhea, with a mortality rate of 42.86%. On the contrary, the group treated with HJ showed a lower incidence of vomiting and diarrhea, with a 100% survival rate (Rao et al., 2023). This suggests that herbal extracts possess antiviral activity against PEDV and could potentially be used as disinfectants in feed and drinking water to reduce morbidity and disease severity in the swine industries (Chen et al., 2022c). The extraction method is a critical step in the preparation of TCM extracts, directly influencing the quality and utilization of active compounds (Bai et al., 2024). Traditional extraction methods generally rely on solvent extraction, using substances such as water, methyl alcohol, ethyl alcohol, and others. The antiviral activity of the extracts acquired through various methods differs. For example, three extracts (PO, PO-P, PO-S) were obtained from *Portulaca oleracea L*. through water extract, alcohol precipitation, and the supernatant after precipitation, respectively. While all three components improved the viability of PEDV-infected VH cells, the PO extract was most effective, with a 92.73% inhibition rate, higher than that of the PO-P (58.61%) and PO-S (80.54%) groups (Liu et al., 2021). Additionally, the polysaccharide content extracted from *Portulaca oleracea* via alcohol precipitation was 52.5%, which was much lower than that obtained using water extraction (Lu et al., 2020). Due to the time-consuming and environmentally concerning nature of traditional extraction methods, novel methods such as supercritical fluid extraction (SFE), microwave-assisted extraction, and ultrasonic-assisted extraction have been explored (Bhadange et al., 2024). Compared to microwave-assisted extraction, SFE and ultrasonic-assisted extractions can be conducted at lower temperatures, making them suitable for heat-sensitive compound extraction, although these methods require expensive equipment and have high operational demands (Christou et al., 2021; Hu et al., 2023). Microwave-assisted extraction significantly reduces extraction time by rapidly heating the solvent and sample, but uneven heating can lead to incomplete extraction (Parappa et al., 2024). Therefore, combining multiple extraction techniques may turn into a trend in the advancement of anti-PEDV TCMs.

**Table 2:** TCM single herb extracts against PEDV.

No.	Name	Source	Main ingredient	Extraction method	IC_50_	TI	Dose or Administration	Virus strain	Mechanism	Effects	Models	References
1	PCP1.1, PCP1.2, PCP2.1, PCP2.2	*Pogostemon cablin*	Polysaccharides	Water extraction and alcohol precipitation	—	—	37.5, 150, 600 μg/mL	CV777	Anti-oxidative	PCP1.1 and PCP1.2 inhibit PEDV replication; PCP2.1 and PCP2.2 inhibit PEDV penetration and replication	IPEC-J2 cells	(Chen et al., 2020c)
2	AOFP3	Fructus of *Alpiniae oxyphyllae*	Polysaccharides	Water extraction and alcohol precipitation	—	—	50, 100, 200, 400, 800 μg/mL	CV777	Anti-oxidative; inhibit the interaction between PEDV S and pAPN proteins; inhibit the activity of PEDV RdRp and hnRNP A1	Inhibit PEDV adsorption and penetration into host cells, and viral replication stage	IPEC-J2 cells	(Chen et al., 2021a; Luo et al., 2022; Wu et al., 2023)
3	—	*Ginkgo biloba* exocarp of yellow color	Polysaccharides	Extract with 98% ethanol at 90°C	1.7±1.3 μg/mL	>58.8	100 μg/mL	CV777	—	Inhibit PEDV attachment and entry steps; inactivate PEDV infection	Vero cells	(Lee et al., 2015b)
4	POP	*Portulaca oleracea* L.	Polysaccharides	Alcohol precipitation	—	—	6.25, 12.5, 25 μg/mL	JX-16	Inhibit the secretion of IFN-α, TNF-α, and IL-6	Inhibit PEDV infection; Inhibit PEDV adsorption	VH cells	(Lu et al., 2020)
5	PO, PO-P, PO-S	*Portulaca oleracea* L.	Polysaccharides	Water extract; alcohol precipitation; the supernatant after precipitation	—	—	6.25, 12.5, 25, 50, 100 mg/mL	JX-16	Decrease the cytokine levels of TNF-α, IL-22 and IFN-α; inhibit the NF-κB pathway	PO inhibit PEDV by 92.73%, while PO-P by 58.61% and PO-S by 80.54%; inhibit PEDV adsorption; effect on PEDV S protein	Vero cells; VH cells	(Liu et al., 2021)
6	—	Stems and leaves of *Pennisetum purpureum*	Polyphenols, flavonoid, etc.	Pulverized extract	—	—	50 μL of 10-, 100-, or 1000-fold diluted extract	Pingtung 52	—	Inhibit PEDV proliferation; infection of PEDV pretreated with a 10-fold dilution of extract for 6 h was 100% inhibited	Vero cells	(Chen et al., 2022c)
7	—	*Aloe vera*	Emodin, etc.	Aqueous extract	—	—	4∼16 mg/mL; 100 mg/kg (in vivo); orally	GDS01	Block transcription of viral N genes and the synthesis of capsid protein	Inhibit PEDV replication; inactivated PEDV directly; do not effect on the viral genome and S1 protein; protect piglets against PEDV infection	Vero cells; IPEC-J2 cells; Piglets	(Xu et al., 2020)
8	—	*Hypericum japonicum*	Flavonoids, etc.	Water extract	—	—	0.0625, 0.125, 0.25, 0.5, 1 mg/mL; 1.28 g/kg (in vivo); orally	CV777; G2	Altered intestinal microbiota composition of piglets	Inhibit PEDV replication, weak inhibitory effect on viral adsorption and invasion stage; protect piglets against PEDV infection	Vero cells; IPI-FX cells; Piglets	(Rao et al., 2023)
9	Compound 1-16	Leaves of *Sabia limoniacea*	Seven new sabphenosides (1-7), a new flavonoid (8),8 known phenolic compounds (9-16)	70% Ethanol extract	7.5 ± 0.7 μM (Compound 15); 8.0 ± 2.5 μM (Compound 16)	—	5, 10, 20, 30 μM	—	Inhibited PEDV S and N protein synthesis; inhibit PEDV 3CLpro	Compounds 7, 8, 11, and 12 showed moderate inhibitory activities, and 15 and 16 exhibited the strongest inhibitory activities	Vero cells	(Cho et al., 2019)
10	Compound 1-13	Radix of *Saposhnikovia divaricata*	Three new (1-3), 10 known coumarins (4-13)	Methanol extract	4.28 ± 0.64 μM (Compound 5)	> 23.90±4.11 (Compound 5)	2.5, 5, 10, 20 μM	—	Inhibit the synthesis of PEDV viral structural protein	Compound 5 revealed strongest inhibitory effect on PEDV replication; Compound 5 effects on GP6 nucleocapsid, GP2 spike, and GP5 membrane proteins of PEDV	Vero cells	(Yang et al., 2015a)
11	MOE	Leaves of *Moringa oleifera*	Quercetin, rutin	Water extract	—	—	500, 1000, 2000 μg/mL	CV777	Anti-oxidative; anti-inflammatory; anti-apoptosis	Inhibit PEDV replication, but not attachment or internalization stage	Vero cells	(Cao et al., 2022)
12	KIOM 198	*Epimedium Koreanum* Nakai	Icariin, quercetin, etc.	Water extract	—	—	0.15, 0.3, 0.6, 0.75, 1.5 mg/mL; 0.6% of diet; orally	KPEDV-9; sm98	Modulate immune response	Inhibit PEDV proliferation; piglets with KIOM 198 treatment did not show any disease symptoms like diarrhea	Vero cells; mouse primary liver cells; Piglets	(Cho et al., 2012)
13	KIOM 124	*Lonicera japonica* Thunberg	—	Water extract	—	—	20-fold diluted (1.5 mg/mL)	KPEDV-9	—	Inhibit PEDV proliferation	Vero cells	(Cho et al., 2012)
14	Compound 1-10	Seeds of *Aesculus turbinata Blume*	Oleanane triterpenoids	Two-step hydrolysis (compound 1-7); n-BuOH extraction (compound 8-10)	—	—	1, 2, 5, 10 μg/mL	—	Inhibit 3CLpro activity of PEDV	Compounds 4 and 6 showed strong inhibitory activities against PEDV	Vero cells	(Kim et al., 2017a)
15	Compound 1-15	Flowers of *Camellia japonica*	Oleanane triterpenes	70% ethanol with sonication	0.28±0.09∼ 0.93±0.22 μM	6.68±0.14∼44.54±8.34	—	—	Inhibit PEDV genes encoding GP6 nucleocapsid, GP2 spike, and GP5 membrane protein synthesis	Compounds 6, 9, 11, and 13 showed most potent inhibitory effects on PEDV replication	Vero cells	(Yang et al., 2015b)
16	10 new and 15 known analogues	Flowers of *Chrysanthemum indicum*	Sesquiterpenoids	95% ethanol at 50°C	—	—	10, 20, 40, 45, 80, 90 μM	—	Compounds 2 and 17 could reduce PEDV N and S protein synthesis	Compounds 1-5, 12, 14, 16, 17, 19, and 21 increased cell viability against cell death	Vero cells	(Liu et al., 2017b)
17	Grape seed extract	Seeds of grape	—	—	2.42 μM	>41.37	2.5, 5, 10 μM	YN150 strain-Nluc; YN150	Decrease ROS production	Inhibition of at least 60% PEDV infection	Vero cells	(Li et al., 2021)

“—” indicates no report

Additionally, to improve resource utilization and reduce costs, extracting active ingredients from waste herbal products is also a promising area of exploration. For example, *Pennisetum purpureum* is not only a medicine source, but can also be applied in knitting, food, paper manufacturing, and biofuel production. In 2022, an extract of *P. purpureum* (Heyiya®), obtained from solid waste byproducts, completely inhibited PEDV in Vero cells when pretreated for 6 h with the a 10-fold dilution. Notably, this extract also displayed inhibitory effects against feline coronavirus (FCoV), PEDV, and IBV, which belong to envelope viruses, as well as EV71, a virus that lacks a lipid envelope (Chen et al., 2022c).

Currently, identifying and analyzing the effective components in herbal extracts is crucial for the modernization of TCM. Studying the material bases, discovering therapeutic or toxic components, and clarifying their targets and mechanisms of action have become key research areas in TCM (Jiang et al., 2022). Using spectroscopic methods and electronic circular dichroism (ECD) spectra analysis, at least a new flavonoid, seven new sabphenosides, three new coumarins, eight known phenolic compounds, and ten known coumarins have been identified from herbal extracts during the search for PEDV inhibitory materials (Cho et al., 2019; Yang et al., 2015a). Among these compounds, one coumarin demonstrated the highest TI of > 23.90±4.11 in Vero cells. Mechanistic studies revealed that the expression of genes encoding PEDV GP6 nucleocapsid, GP2 spike, and GP5 membrane proteins were inhibited by this compound in a dose-dependent manner, providing a foundation for the development of new chemical entities against PEDV (Yang et al., 2015a). However, many bioactive compounds in herbal extracts that exhibit inhibitory effects against PEDV infection remain unidentified (Cao et al., 2022; Cho et al., 2012; Rao et al., 2023). The challenges in confirming active ingredients are significant, including complex composition, multi-target actions, interactions between components, variation in extraction methods, and the lack of uniform standards (Jiang et al., 2022). To address these challenges, various technologies such as liquid chromatography-mass spectrometry (LC-MS), nuclear magnetic resonance (NMR) (Wolfender et al., 2019), selected reaction monitoring (SRM) (Song et al., 2017), network pharmacology (Yang et al., 2021a), have been developed. These advanced technologies will facilitate the rapid identification of complex components in TCM in the future.

### 4.3 TCM recipes

In combating various viral diseases, TCM has developed a solid theoretical system and profound clinical experience for controlling “pestilence” (Liu et al., 2017a). Based on TCM theory, classical recipes typically consist of multiple herbs or minerals, with one serving as the principal ingredient, while the others acting as adjuvants to enhance the effects or improve the delivery of the principal ingredient (Chen et al., 2020b). For example, *Pulsatilla* Powder, a classic prescription known for its detoxifying and dysentery effects, is primarily based on *Pulsatilla*, supplemented with *Coptidis rhizome* and *Cortex phellodendri chinensis*, and has been used clinically to treat diarrhea for hundreds of years (Yu et al., 2017). Recently, 11-day-old piglets were subjected to PEDV (HN2021 strain) at a dose of 10^4.5^ TCID_50_ per pig, and the *Pulastilla* Powder decoction was administered orally for five days (0.2 mL/kg/day). After treatment, the replication of the PEDV M gene in the intestine tissues of PEDV-infected piglets was inhibited, and the diarrhea rate was also reduced (Chang et al., 2023). One of the key reasons for the effective PEDV treatment was that *Pulsatilla* Powder contains a variety of active ingredients, including *Pulsatilla* saponin, berberine, and palmatine hydrochloride, all of which have antibacterial, antiviral, and immunomodulatory effects (Yu et al., 2017). Evidence suggests that probiotic fermentation of TCMs can significantly modify the ingredients and create new bioactive components with medicinal value (Guo et al., 2024). To further improve its therapeutic effect, *Pulsatilla* Powder was fermented by *Lactobacillus casei* R. As expected, the fermentation liquid exhibited better treatment results in piglets than the decoction, suggesting that fermentation released the active ingredients more effectively. Furthermore, *Lactobacillus casei* may help regulate intestinal microbiota, contributing to a reduction in diarrhea (Chang et al., 2023). Thus, regulating gut microbiota could be an effective strategy for preventing and treating PED. Similarly, several other classic TCM prescriptions, such as Shenling Baizhusan, Gegen Qinlian decoction, Xiaojian Zhong, and Jingsananli-sepsis, have also effectively reduced the diarrhea rate in piglets by relieving inflammation and improving the intestinal environment (Chen et al., 2020b; Liu et al., 2017a; Liu et al., 2019). However, it is still unclear whether these prescriptions can inhibit the PEDV infection, which warrants further investigation.

In many cases, TCM recipes are tailored to the symptoms of individual patients or animals (Huang et al., 2021b). Kim et al. investigated the anti-PEDV effects of a combined mixture of extracts from four medicinal herbs (*Taraxaumi mongolicum, Viola yedoensis Makino, Rhizoma coptidis*, and *Radix isatidis*), named MYCI. Newborn piglets were orally administrated MYCI (60 mg/day) for 7 days following PEDV infection. The MYCI-treated group exhibited a significantly higher daily weight gain compared to the vehicle group (7 g vs. -18 g). More importantly, the severe atrophy of long villi and crypt hyperplasia induced by PEDV infection were alleviated by MYCI treatment (Kim et al., 2015). Therefore, the MYCI mixture may be a promising anti-PED drug. However, the diarrhea score and fecal PEDV shedding remained unaffected by MYCI treatment (Kim et al., 2015). It would be worth exploring whether combining the PEDV vaccine with MYCI would have any synergistic preventive effects against PED. Furthermore, other self-made or modified TCM recipes described in Chinese literatures have contributed to PED treatment *in vivo* (Table 3), including the *Andrographis paniculata* compound (Liu and Zhai, 2022) and other compound Chinese herbal medicines (Yaxin et al., 2020), which have shown high cure rates of over 50%. These results suggest that TCM prescriptions may offer a potential therapeutic strategy for piglet diarrhea.

**Table 3:** TCM recipes against PEDV.

No.	Chinese medicine compound	Components composition (Latin names)	Virus strain	Dose or Administration	Mechanism	Effects	Models	References
1	*Pulsatilla* powder	*Phellodendri chinensis Cortex, Coptis chinensis Franch, Pulsatillae chinensis Regel, Fraxini Cortex, etc.*	HN2021	0.2 mL/kg, 5 days; orally	Regulate intestinal microbial flora; reduce oxidative damage	Inhibit PEDV proliferation; increase ADG of piglets; decrease the diarrhea rate; alleviate intestinal pathological damage	Piglets	(Chang et al., 2023)
2	Andrographis paniculata compound	*Andrographis paniculata Nees, ficus carica Linn, Folium lsatidis, Portulaca oleracea Linn, etc.*	CV777	2 mL/kg, 2 times/day, 3 days; orally	Stimulate the TLR3/IFN-β signaling pathway	100% treatment rate, 50% cure rate for PEDV-infected piglets	Piglets	(Chang et al., 2023)
3	Chinese herbal medicine	*Houttuynia cordata Thunb, Coptis chinensis Franch, Taraxacum mongolicum Hand-Mazz, Isatis tinctoria L, Astragalus membranaeus, Radix Pseudostellariae.*	—	0.4, 0.8, and 1.2 g/day; orally	Enhance growth performance and intestinal health of piglets	96.7% survival rate; increase ADG of piglets; alleviate intestinal pathological damage	Piglets	(Liu and Zhai, 2022)
4	Chinese herbal medicine	*Pulsatillae chinensis Regel, Scutellariae baicalensis Georgi, Fraxini Cortex, Codonopsis pilosula, Atractylodes lancea, Glycyrrhiza uralensis Fisch, Angelica sinensis, Cinnamomum cassia Presl, Magnolia officinalis Rehd. et Wils, Terminalia chebuta Retz, Crataegus pinnatifida Bge.*	—	0.75 mL/kg, 2 times/day; orally	Protect mitochondria; stabilize the structure of gastrointestinal mucosa; regulate the activity of enzymes	Alleviate intestinal pathological damage	Piglets	(Yaxin et al., 2020)
5	MYCI	*Taraxaumi mongolicum, Viola yedoensis Makino, Rhizoma coptidis, Radix isatidis*	—	60 mg/day, 1 mL, 7 days; orally	Enhance growth performance and intestinal health of piglets	Increase ADG of piglets; decrease the diarrhea rate; ameliorated intestinal lesion of newborn piglets	Piglets	(Kim et al., 2015)
6	Chinese herbal medicine	*Atractylodes macrocephala Kodiz, Codonopsis pilosual (Franch) Nannf, Radix Aucklandiae, Poria cocos, Haematium, Terminalia chebuta Retz.*	—	60 mL; 2 times/day; orally	Enhancegrowth performance of piglets	Increase effective treatment rate for PEDV-infected piglets	Piglets	(Yang et al., 2019)
7	Chinese herbal medicine	*Andrographis paniculata (Burm. F.) Nees, ficus carica Linn, Folium lsatidis, Portulaca oleracea Linn, Astragalus membranaeus, etc.*	CV777	0.2 and 0.4 mL/kg, 2 times/day, 3 days; orally	Enhance growth performance of piglets	50∼60% treatment rate, 30∼50% cure rate for PEDV-infected piglets	Piglets	(Liu et al., 2016)

“—” indicates no report.

### 4.4 Anti-PEDV mechanisms of TCM

#### 4.4.1 Inhibition of viral life cycle

**Fig. 3:**
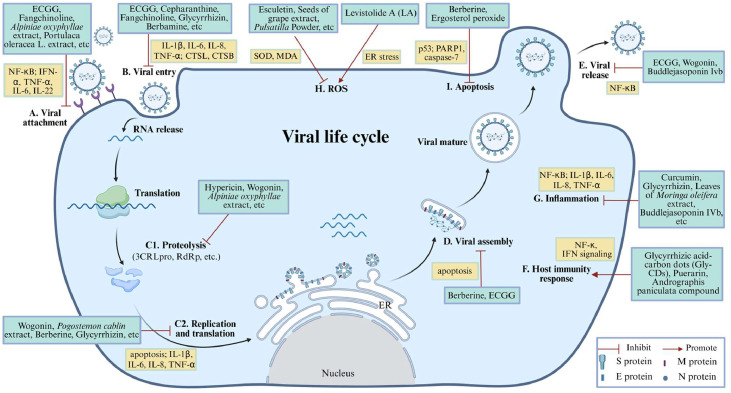
The summary of anti-PEDV mechanisms of different TCMs. TCMs play an antiviral role by inhibiting different stages of viral life cycle, and the key stages including viral attachment (A), viral entry (B), proteolysis, replication and translation (C), viral assembly (D) and viral release (E) were labeled from A to E, respectively. The antiviral modes of TCMs also by regulating the modulation of host factors, such as host immunity response (F), inflammation (G), ROS (H) and apoptosis (I).

Three essential steps occur throughout the virus life cycle: viral attachment and entry, viral replication, and viral assembly and release (Li and Peng, 2013). To date, many kinds of TCM have been confirmed to control PEDV (Tables 1-3). To investigate the specific stage of the virus life cycle at which the compounds exert their more efficient effects, the administration protocols of the compounds and the corresponding viral-cycle stages that might be inhibited were designed as follows: for -1 hpi-treatment, attachment; for 0 hpi-treatment, attachment, and entry; for virus-pretreatment, interaction of compounds and virus; for 4°C attachment-treatment, entry; and for 2 hpi-treatment, virus replication (Dong et al., 2022). The results showed that fangchinoline more efficiently exerted its inhibitory effects by interrupting the virus attachment and entry stages, or by attenuating the virus directly, while cepharanthine and tetrandrine mainly effect on virus entry (Fig. 3) (Dong et al., 2022). Likewise, another bis-benzylisoquinoline alkaloid, berbamine, also inhibited PEDV entry, but did not affect viral adsorption (Zhang et al., 2023a). Overall, these compounds could be considered as lead compounds against PEDV.

Viral replication is the core of the viral life cycle, and most current antiviral agents target this stage (Li and Peng, 2013). As we know, multiple proteases and replicates are encoded in coronaviruses, such as 3C-like protease (3CLpro) and RNA-dependent RNA polymerase (RdRp), which are indispensable in viral replication, making them promising targets for antiviral research (Liu et al., 2022). Quercetin, a flavonol metabolite of *Bupleurum chinense* DC., possesses antioxidative (Robaszkiewicz et al., 2007), anticancer (Dajas, 2012), antibacterial (Li and Xu, 2008), and antiviral effects (Choi et al., 2009b). Based on molecular docking studies, surface plasmon resonance (SPR) analysis, and the fluorescence resonance energy transfer (FRET) assay, quercetin exhibited a binding affinity and inhibitory effect on PEDV 3CLpro, with an IC_50_ of 1.7±0.8 μg/mL against the PEDV CV777 strain (Li et al., 2020d). Interestingly, quercetin 7-rhamnoside showed higher anti-PEDV activity with an IC_50_ of 0.014±0.005 μg/mL, indicating that sugars groups at C-7 of the A-ring are important against PEDV, which may be useful for designing other related antiviral agents (Choi et al., 2009a). Additionally, another flavonoid compound wogonin (Wang et al., 2023a), phenol compound hypericin (Zhang et al., 2021b), and alkaloid compound tomatidine (Wang et al., 2020a) also showed inhibitory effects on PEDV 3CLpro. These research results indicate that 3CLpro is a promising target against PEDV. However, further animal studies are still needed to investigate the anti-PEDV effect.

Last but not least, several natural compounds showed inhibition of viral assembly and release. For example, berberine, an alkaloid metabolite from *Coptis chinensis* Franch., has anti-PEDV activity in Vero cells, inhibiting the replication and assembly stages of PEDV (Ye et al., 2019). Moreover, wogonin not only affects PEDV replication by targeting 3CLpro, but also affects PEDV release, potentially through the regulation of innate immune responses (Wang et al., 2023a). Additionally, a natural compound in green tea, epigallocatechin-3-gallate (EGCG), making up approximately 59% of its total catechin content. A recent report demonstrated that EGCG prevented PEDV infection in a dose-dependent manner in Vero cells (IC_50_=12.39∼83.18 μΜ) (Huan et al., 2021), showing similar antiviral activity against porcine circovirus type 2 (PCV2) with an IC_50_ was 37.79±1.64 μΜ (Li et al., 2020a). Moreover, EGCG exerted its anti-PEDV effects by preventing viral attachment, entry, replication and assembly (Huan et al., 2021). EGCG also exhibited inhibition of human immunodeficiency virus (HIV) and PRRSV (Fassina et al., 2002; Ge et al., 2018). However, the poor bioavailability (about 4.5∼7.2%) restricts its clinical application (Del Rio et al., 2010). Therefore, further study is required to increase its bioavailability.

#### 4.4.2 Anti-inflammatory

Inflammation is closely associated with many diseases, such as tumors and cardiovascular disease, and is a key factor in the pathogenesis of diseases (Guo et al., 2015). It is reported that PEDV infection leads to the acetylation and release of high mobility group box1 (HMGB1), which promotes the release of proinflammatory cytokines (Huan et al., 2016). During PEDV infection, a variety of proinflammatory cytokines, including IL-1β, IL-6, IL-8, TNF-α, and chemokines (such as MCP-1) are produced by host cells to initiate immune responses *in vitro* and *in vivo* (Annamalai et al., 2015; Huan et al., 2017; Wu et al., 2020). While low levels of proinflammatory cytokines can protect against viral invasion, an overproduction of cytokines can impair the host immune response, even leading to more serious disease (Broggi and Granucci, 2015). Therefore, these proinflammatory mediators have a significant impact on inflammatory and disease progression (Peterson et al., 2001). TCM with anti-inflammatory effects can defend against viral infections by alleviating the inflammatory response. For example, as a competitive inhibitor of HMGB1, glycyrrhizin, the primary constituent in licorice root extracts, could lead to a roughly 70% decrease in viral ORF3 gene expression at 0.8 mM concentration when the multiplicity of infection (MOI) was 0.1, and decrease the mRNA levels of proinflammatory cytokines at 6, 12 and 24 hpi in PEDV-infected Vero cells: IL-1β (20%, 49%, 75%), IL-6 (39%, 53%, 80%), IL-8 (46%, 47%, 94%), and TNF-α (51%, 56%, 91%) (Huan et al., 2017). Moreover, glycyrrhizin has also been used for treating ulcerative colitis and porcine endotoxemia owing to its anti-inflammatory properties (Yuan et al., 2006). Flavonoids have biological functions such as antibacterial, anti-inflammatory, antiviral, antioxidant, immunomodulatory, and anti-tumor activities, and also play an important role in the process of anti-PEDV infection. Among these, puerarin, an active isoflavone in the traditional Chinese medicinal plant *Pueraria* species such as *P. lobata* (Wild) *Ohwi*, and *P. mirifica*, has anti-inflammatory activities (Xu et al., 2024). Excitingly, puerarin not only reduced PEDV replication in Vero cells and the mRNA amounts of PEDV M and N genes, but also helped restore growth performance of PEDV-infected piglets. Further studies found that puerarin downregulated the expression levels of cytokines (including IL-8, IL-6, and IL-1β) in the ileum in PEDV-infected piglets. Moreover, both NF-κB and pNF-κB expression *in vivo* were brought back to control levels via puerarin treatment by Western blot assay, revealing that puerarin can regulate the NF-κB signaling pathway (Wu et al., 2020). These results indicate the potential for developing glycyrrhizin and puerarin as anti-PEDV agents.

#### 4.4.3 Enhancement of the host immune response

IFN is one of the most important parts of innate immunity, playing an essential role in antiviral activity (Schoggins et al., 2011). PEDV has the ability to interrupt both the induction of host IFN and the function of antiviral interferon-stimulating genes (ISGs) (Wang and Zhang, 2023). TCMs have been proven to regulate the function of the host immune system by modulating the number and activity of immune cells and molecules, improving the antiviral ability of the host. For instance, curcumin, an active polyphenol compound extracted from the rhizome of *turmeric*, exhibits various biological functions such as anti-tumor, antibacterial, and antiviral properties (Lao et al., 2006). Liu et al. reported that curcumin could inhibit the proliferation of PEDV in Vero cells, partly by increasing the expression of antiviral cytokines, including IFN-α and IFN-β (Liu et al., 2023). Additionally, curcumin has been confirmed to have the inhibition effect for HIV (Jennings and Parks, 2020), HPV (Maher et al., 2011), TEGV (Li et al., 2020c), and PRRSV (Du et al., 2017). Puerarin could inhibit PEDV infection through multisite inhibition mechanisms, including regulating the IFN signaling pathway. Puerarin treatment could decrease the expression levels of IFN-α and IFN-β in piglets with PEDV infection (Wu et al., 2020). Glycyrrhizic acid has various biological activities but is limited by poor water solubility and certain cytotoxicity (Størmer et al., 1993). Carbon dots (CDs) are carbon nanomaterials with small sizes (d<10 nm) and controllable surface functional group, which have unique properties and biocompatibility and have been extensively applied (Yang et al., 2019). Subsequently, glycyrrhizic-acid-based carbon dots (Gly-CDs) with high dispersibility and small size were synthesized, and it was found that Gly-CDs could significantly inhibit several virus infections, including PEDV, PRRSV, and pseudorabies virus (PRV) (Tong et al., 2020). Notably, after MARC-145 cells were incubated with Gly-CDs at a concentration of 0.30 mg/mL for 24 h, a significant increase in the mRNA expression levels of ISGs, including ISG-54, ISG-56, ISG-60, oligoadenylate synthetase (OAS), and zinc finger antiviral protein (ZAP) was detected (Tong et al., 2020). These ISGs are known to positively modulate the expression of phosphorylated signal transducer and activator of transcription 1/IFN-β/retinoic acid-inducible gene G to inhibit viral infection, thereby inhibiting viral infection and inducing pro-inflammatory reactions (Bréhin et al., 2009; Schoggins et al., 2011; Zhu et al., 2014). These findings suggest that Gly-CDs may suppress PEDV infection via stimulating the innate immune signaling pathways, such as IFN pathway.

#### 4.4.4 Modulation of reactive oxygen species (ROS) levels

Reactive oxygen species (ROS) are widely thought to be signaling molecules that participate in various physiological processes, but excess ROS can lead to oxidative stress (OS) (Harshithkumar et al., 2024). Previous studies revealed that PEDV infection caused an accumulation of ROS in Vero cells in a time-dependent manner (Liu et al., 2022; Xu et al., 2019). Subsequently, ROS accumulation was a key factor for endoplasmic reticulum (ER) stress and autophagy (Sun et al., 2021). Therefore, antioxidants that suppress the ROS production may become promising prevention and treatment drugs for PEDV infection. Interestingly, out of 25 compounds with anti-PEDV activity identified from a panel of 803 natural product extracts, seven are antioxidants, including betulonic acid, ursonic acid, esculetin, lithocholic acid, nordihydroguaiaretic acid, caffeic acid, and grape seed extract. Significantly, all the 7 antioxidants still showed efficient inhibition of at least 60% in Vero cells at a concentration of 2.5 µM. Further studies found that PEDV-induced ROS production was obviously deceased in cells treated with these antioxidants (Li et al., 2021). Similarly, polysaccharide AOFP3 isolated from *Alpiniae oxyphyllae* fructus could protect IPEC-J2 cells from PEDV infection while improving the antioxidant activity of the cells (Chen et al., 2021a). However, conversely, another PEDV inhibitor, Levistolide A, an important active component exists in *Ligusticum chuanxiong*, can induce ROS generation and stimulate ER stress. Moreover, inhibiting ROS production with the antioxidant N-Acetylcysteine (NAC) could counteract the antiviral activity of Levistolide A (Zeng et al., 2022b). These studies indicated that ROS can activate host antiviral responses but may also boost viral replication (Harshithkumar et al., 2024). Cells can benefit from moderate levels of ROS, including enhanced proliferation, metabolic regulation, physiological functions, and defense against pathogens (Mittler, 2017). In addition, the inconsistent results may also be related to differences in drugs, dose used, and virus strains, requiring further research to explore the underlying mechanisms.

#### 4.4.5 Modulation of apoptosis

Apoptosis is a programmed cell death to maintain the homeostatic balance between cell proliferation and cell death, but the disruption of this balance can lead to abnormal physiological activities and diseases. It has been documented that PEDV can cause apoptosis by activating mitochondrial apoptosis-inducing factor (Kim and Lee, 2014) and/or activating caspase-8 and caspase-3 during the late phase of PEDV infection (Chen et al., 2018; Zhang et al., 2019b). The apoptosis induced by PEDV infection contributes to cytopathic effects (CPE) *in vitro* and tissue damage *in vivo*, thus interfering with the absorption of nutrients and water, leading to diarrhea in piglets (Duan et al., 2021; Ren et al., 2020; Zhang et al., 2021a). Ergosterol peroxide (EP) is a sterol with multiple bioactivities including antiviral effects on several human and animal viruses (Huang et al., 2021a). In 2022, Liu et al. found that EP, derived from the fruiting structure of the mushroom *Cryptoporus volvatus*, significantly inhibited the expression level of PEDV N protein in a dose-dependent manner (reduction rate: 30.1∼82.6%) in Vero cells. Additionally, EP treatment mitigated PEDV-induced apoptosis (Liu et al., 2022). In light of the p53 pathway is a classical pathway of apoptosis (Chipuk et al., 2004), p53 inhibitor pifithrin-α (PFT-α) was used to investigate the connection between p53 and PEDV infection. The result showed that PFT-α significantly decreased the cell apoptosis, and the expression of PEDV N protein, cleaved caspase-3, Bax, increased Bcl-2 expression in PEDV-infected cells, implying that p53 play a key role in viral replication and apoptosis induced by PEDV (Liu et al., 2022). In addition, p53 can be regulated by ROS, which functions as an upstream signal to regulate the PEDV-induced apoptosis (Liu et al., 2022; Xu et al., 2019). Therefore, EP can inhibit apoptosis induced by PEDV via interfering the ROS-dependent p53 signaling pathway. Another study found that the marker proteins of apoptosis (such as PARP1 and caspase-7) were downregulated in the cells with berberine treatment, thereby berberine could suppress PEDV-induced apoptosis (Ye et al., 2019).

## 5 Current challenges and prospects

### 5.1 Current challenges of TCM

Over the past 3000 years of Chinese history, TCM has been routinely used to treat pandemic diseases, showing exciting results in managing infectious diseases (Huang et al., 2021b). Up to now, TCM remains an irreplaceable part of medical treatment for both humans and animals. For example, Professor Tu Youyou (Ma et al., 2020) drew inspiration from the ancient Chinese medicine text Ge Hong's Zhouhou Beiji Fang, leading to the development of artemisinin for treating malaria, for which she won the Nobel Prize. In addition, TCM has made a significant contribution to the 2019 novel coronavirus disease (COVID-19) control (Zhao et al., 2021). A clinical trial involving 123 suspected and confirmed COVID-19 patients found that treatment of Jinhua Qinggan granules, combined with antiviral western medicine, helped relieve clinical symptoms such as fever and poor appetite, and antibiotic use was significantly lower compared to the group treated with only antiviral western medicine(An et al., 2021). Furthermore, a recent study found that oral Huoxiang suling shuanghua decoction (HSSD) could enhance the immunogenicity of the SARS-CoV-2 inactivated vaccine (Tang et al., 2024). This suggests that TCM can not only have antiviral effect but also work synergistically with conventional antiviral drugs or vaccines to improve efficacy. However, the clinical application of TCM faces challenges. TCM treatment typically involves long course of therapy, and its effectiveness is hindered by factors such as the single approach to administration, low bioavailability, limited biological half-life, poor targeting effect, rapid metabolism (Wei et al., 2022), and potential toxic side effects (Yang et al., 2023), which restrict its clinical application to a great extent (Peng et al., 2024; Yang et al., 2023). Therefore, more research is needed to explore approaches such as nano-TCM and fermented TCM to enhance its efficacy in controlling PEDV.

### 5.2 Enhancement of therapeutic effect of TCM

There are various strategies to enhance the therapeutic efficacy of drugs, including optimizing new drug design, improving drug delivery systems, monitoring drug preparation and effects, and utilizing drug combination therapies (Kesik-Brodacka, 2018). One promising approach is nano-TCM, which involves integrating TCM with nanotechnology to create novel and effective drug formulation. These nano-TCM formulations, typically ranging from 1 to1000 nm in size, offer advantages such as a large surface area, strong targeting ability, sustained release, higher bioavailability and stability, and minimal side effects, which can address several obstacles encountered by conventional drugs in treating diseases (Almeida et al., 2020; Liu and Feng, 2015). For example, Tong et al. synthesized a highly biocompatible carbon dot (Gly-CD) from the active ingredient glycyrrhizic acid using a hydrothermal method. Gly-CDs were shown to significantly inhibits the proliferation of PRRSV and reduce the theoretical side effect of glycyrrhizic acid (Tong et al., 2020). Thus, nanotechnology is emerging as a research hotspot in TCM. Nanotechnology applied to TCM can take two forms. (1) Converting TCM drugs into nanoscale particles through methods like mechanical crushing, spray drying, and high-pressure homogenization(Wei et al., 2022). For example, the crude realgar possessed slight cytotoxicity to cancer, while realgar nano-grinded with a diameter of 100∼200 nm showed robust inhibition effect in cancer cell lines (Yang et al., 2021b; Zhao et al., 2011). (2) Using nanocarriers, such as liposomes, microemulsions, extracellular vesicles, and polymeric nanoparticles (PNPs), for drug delivery (Wei et al., 2022). Wang et al. prepared berberine nanoliposomes using PEGylated liposomes with low toxicity and biodegradability, and then modified with lactoferrin for brain-targeting. Based on a mouse model of Alzheimer's disease, they found that lactoferrin-modified berberine nanoliposomes improved bioavailability and neuroprotective effects of berberine (Wang et al., 2023b). Therefore, combining PEDV prevention and treatment drugs with a nano-delivery system could offer promising therapeutic effects. While a variety of nano-TCM formulations have been developed, each preparation has limitations. Additional research is necessary to explore the theory, safety, efficacy, stability, and mechanisms of nano-TCM preparation, and more extensive clinical trials are required (Peng et al., 2024).

Fermentation of TCM is another essential technique (Wang et al., 2024). Modern TCM fermentation technology is categorized into liquid fermentation and solid fermentation, depending on the state of the fermentation medium (Wu et al., 2020). Under appropriate conditions of temperature, humidity, and moisture, fermentation can enhance the original properties of TCM (Chang et al., 2023) and produce new therapeutic effects (Okamoto et al., 2020; Xu et al., 2023), reducing side effects (Kun et al., 2017; Qu et al., 2023). Therefore, the combination of probiotics and TCM shows potential for enhancing the modern application of herbal fermentation. In the context of TCM fermentation, the selection of fermentation strains can be divided into two main types: single-strain fermentation (Li et al., 2020b) and mixed-strain fermentation (Qiufeng et al., 2023). In animal husbandry, using probiotics to ferment TCM can improve growth performance, regulate gut microbiota, enhance palatability, and reduce costs. A recent study showed that fermented *Pulsatilla* Powder had a better treatment effect on piglets infected with PEDV compared to decoction (Chang et al., 2023). However, optimizing the fermentation process remains a challenge, as various parameters like temperature, humidity, pH levels, can significantly affect fermentation efficiency, making it difficult to control the quality of the final product (Wang et al., 2024). Rigorous and systematic quality control standards are essential to ensure safety and effectiveness of fermented TCM. With the above safeguards, the application of probiotics-fermented TCM holds significant potential in healthcare and animal husbandry.

### 5.3 Chiastopic fusion of artificial intelligence (AI) and TCM

Natural products that extracted from plants, fungi, and bacteria offer great potential for antiviral prevention and treatment. Compounds such as quercetin (Li et al., 2020d), quercetin 7-rhamnoside (Choi et al., 2009a), glycyrrhizin (Huan et al., 2017), and surfactin (Yuan et al., 2018) have been demonstrated to inhibit PEDV. Traditionally, the efficacy evaluation of natural compounds against PEDV has involved infection studies, RT-PCR detection, and viral titer quantification. However, these methods are labor-intensive, time-consuming, which poses challenges for high-throughput screening (HTS). Integrating a reporter gene into viral genome can provide a faster, more effective way for monitoring viral replication, making it suitable for HTS and the evaluation of antiviral drugs. To date, several reporter CoVs have been genetically engineered to achieve more effective screening of anti-CoV compounds. For example, a library of 2000 compounds were screened using a recombinant human CoV OC43 (HCoV-OC43) that expresses *Renilla* luciferase, identifying 36 inhibitors targeting HCoV-OC43 replication *in vitro* (Shen et al., 2019). In 2021, Li et al. developed a reverse genetics platform for PEDV (YN150 strain), and established a reporter PEDV expressing nano-luciferase (NLuc) (rPEDV-NLuc). This platform provides a powerful tool for screening and evaluating anti-PEDV compounds (Li et al., 2021).

With the rapid development of artificial intelligence (AI) technology, more and more researchers have applied AI in drug discovery and repurposing in recent years (Wan et al., 2025). The ITCM platform, the largest online drug transcriptomics platform to date for screening TCM active ingredients, using computational screening based on gene expression profiles to facilitate drug discovery (Tian et al., 2023). This approach offers a low-cost, accurate method for identifying effective ingredients, providing a solid theoretical foundation for TCM development. Meanwhile, the powerful data-mining, computing and learning ability of AI technology also greatly speed up the development of herb quality standardization(Pan et al., 2024), drug target discovery, optimized compatibility (Li et al., 2022a), and medical diagnoses, and precise medical care(Zhou et al., 2024), which bring unprecedented opportunities to TCM. However, currently, the integration of TCM and AI is still in its infancy and faces many challenges, such as database quality issues, lack of humanistic care(Zhang et al., 2023c; Zhou et al., 2024). The biggest challenge for AI is the need for large datasets to generate credible predictions, meaning it still has a long way to go before fully supporting scientific research in the future. Nevertheless, the combination of TCM and AI is an inevitable trend, and has exciting prospects (Li et al., 2023b).

### 5.4 Development of novel antiviral drug

A current focus in antiviral drug research is targeting host factors that are essential for viral replication. These host-targeting antiviral drugs offer broad-spectrum efficacy and low resistance (Malone et al., 2022). For example, pyrimidine bases are essential for viral replication, and inhibitors targeting host pyrimidine synthesis have become a significant focus for developing broad-spectrum antiviral drugs (Farghaly et al., 2023). In particular, inhibitors of human dihydroorotate dehydrogenase (hDHODH) have demonstrated broad-spectrum antiviral effects by inhibiting viral replication and modulating immunity, and several hDHODH inhibitors are currently in clinical trials for COVID-19 (Calistri et al., 2021). Another promising avenue involves 3CLpro inhibitors, preventing viral replication by inhibiting the 3CLpro protease activity (Al Adem et al., 2024). For instance, Ye et al. (Ye et al., 2020) used GC376 to block the catalytic residues of 3CLpro, inhibiting the replication of CV777 and YN144 virus strains in cell experiments. Pfizer's Paxlovid (nirmatrelvir/ritonavir) (Hammond et al., 2022) and Shionogi's S-217622 (Unoh et al., 2022) are both 3CLpro inhibitors that have shown good antiviral effects in clinical trials. Additionally, inhibitors targeting the RdRp, such as molnupiravir (Huang et al., 2023), disrupt viral RNA replication. These advancements demonstrate the diversity and innovation in antiviral drug development, offering broader treatment options to combat viral mutations and future pandemics.

## 6 Conclusions

Conclusively, both monomers and extracts from single herb, as well as TCM recipes, exhibit significant anti-PEDV activity by influencing the viral life cycle and host factors. Particularly, TCM recipes and extracts are complex mixtures that contain a variety of active ingredients, which can act on multiple stages of the viral life cycle simultaneously. Compared to chemically synthesized drugs, TCM monomers and extracts generally have lower toxicity and fewer side effects, making them more suitable for long-term use. Additionally, TCM can regulate the host's immune system, enhancing both innate and adaptive immune responses to viruses. While combating viral infections, TCM also exerts anti-inflammatory effects, alleviating inflammation caused by viral infections. Although there are some current challenges, such as the complexity of TCM ingredients leading to unclear mechanisms of action, the complexity of extraction and purification processes, and significant challenges in quality control and standardization, further research is needed to better understand and harness the advantages of TCM. We believe that TCMs holds great promise in controlling PEDV. Furthermore, novel strategies for the development of highly effective vaccines and antibody drugs should be pursued in future research. The cross-integration of biotechnology, information technology, material science, and traditional antiviral drugs will offer great potential and diverse development directions.

## Abbreviations

The following abbreviations are used in this manuscript:

**Table:** 

DCs	Dendritic cells
E	envelope protein
EGCG	epigallocatechin-3-gallate
Gly-CDs	glycyrrhizic-acid-based carbon dots
HJ	*Hypericum japonicum*
HTS	high-throughput screening
IFNs	interferons
IgY	immunoglobulin yolk
ISGs	IFN-stimulated genes
M	membrane protein
MDA	malondialdehyde
m6A	methylation of the N6 position of adenosine
N	nucleocapsid protein
NSPs	nonstructural proteins
ORFs	open reading frames
PCV2	porcine circovirus type 2
PED	porcine epidemic diarrhea
PEDV	porcine epidemic diarrhea virus
PRRSV	porcine reproductive and respiratory syndrome virus
RdRp	RNA-dependent RNA polymerase
ROS	reactive oxygen species
S	spike protein
SOD	superoxide dismutase
TCMs	traditional Chinese medicines
TGEV	transmissible gastroenteritis virus
3CLpro	3C-like protease

## Funding information

Supported by the grants from the Horizontal Cooperation Project of Henan Province University-Enterprise (No. 20200013), the Key Research Projects of Higher Education Institutions of Henan Province of China (No. 23A180014), College Student Innovation and Entrepreneurship Training Program Project of Henan Province of China (No. 202310471033), Research Nursery Project of Henan University of Chinese Medicine (No. MP2024-55).

## Data availability

No data was used for the research described in the article.

## CRediT authorship contribution statement

**Conghao Ji:** Writing – original draft, Methodology, Formal analysis. **Shuxuan Li:** Writing – original draft, Methodology, Funding acquisition, Formal analysis. **Cunhai Hu:** Data curation. **Tongtong Liu:** Data curation. **Qingqing Huang:** Data curation. **Mengyuan Yang:** Data curation. **Mengxin Yang:** Data curation. **Qianqian Wang:** Writing – review & editing, Validation. **Aifang Li:** Writing – review & editing, Validation. **Dandan Guo:** Writing – review & editing, Validation. **Yu Huang:** Writing – review & editing, Validation. **Sugai Yin:** Writing – review & editing, Validation. **Shuying Feng:** Writing – review & editing, Supervision, Funding acquisition.

## Declaration of competing interest

The authors declare that they have no known competing financial interests or personal relationships that could have appeared to influence the work reported in this paper.

## Data Availability

No data was used for the research described in the article.
